# Genes for regeneration

**DOI:** 10.7554/eLife.02517

**Published:** 2014-04-15

**Authors:** Janet Rossant

**Affiliations:** 1 **Janet Rossant** is an *eLife* senior editor and is at the Hospital for Sick Children Research Institute, University of Toronto, Toronto, Canadajanet.rossant@sickkids.ca

**Keywords:** planaria, stem cells, regeneration, single-organ regeneration, pharynx, other

## Abstract

*FoxA*, an evolutionarily conserved gene involved in the development of the digestive system in many animals, has an important role in regeneration in flatworms.

**Related research article** Adler CE, Seidel CW, McKinney SA, Sánchez Alvarado AS. 2014. Selective amputation of the pharynx identifies a FoxA-dependent regeneration program in planaria. *eLife* **3**:e02238. doi: 10.7554/eLife.02238

**Image** A planarian with its pharynx extended

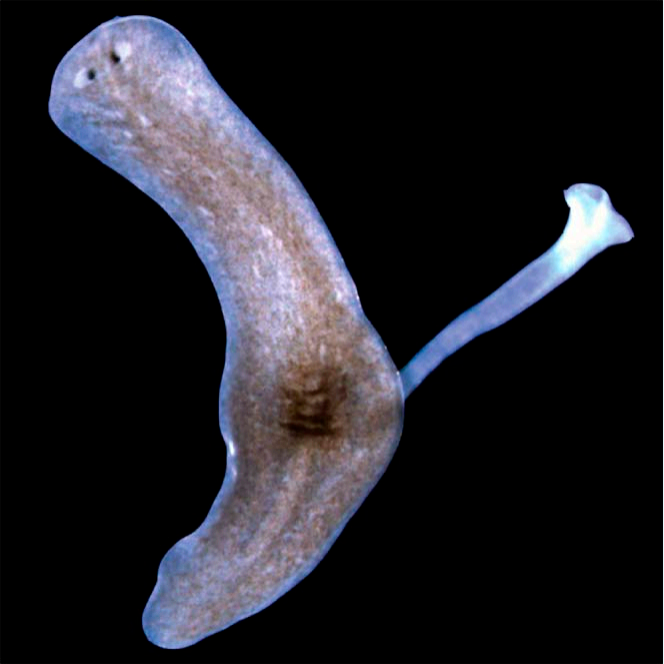

Planarian flatworms are well known for their amazing regenerative capacity. In a manner reminiscent of the Sorcerer’s Apprentice, chopping one worm into little pieces will result in a dish full of tiny worms regenerated from the fragments in just a few days. In recent years this system has been rediscovered as an experimental model for probing how and why tissues regenerate, with the hope that this will help us to improve tissue repair in our own bodies.

Regeneration in planarians depends on the presence of stem cells called neoblasts. These cells are distributed throughout the body and, when part of the worm has been amputated, they are activated to reform the tissues that have been removed (Wagner et al., 2011). It is still not entirely clear how the stem cells regenerate specific organs. Are there different types of stem cell that form different tissues? Do signals produced by nearby cells cause specific tissues to form? Or is a combination of both stem cell bias and local signalling used? Now, in *eLife*, Alejandro Sánchez Alvarado and colleagues at the Stowers Institute for Medical Research—including Carolyn Adler as first author—have provided new insights into this question by developing a method to specifically remove the pharynx, the feeding organ of the worm, to study organ-specific regeneration (Adler et al., 2014).

The pharynx itself contains no stem cells and cannot regenerate the rest of the worm (Figure 1). However, a worm without a pharynx can rapidly regenerate this rather complex organ. Adler et al. found that incubating flatworms in sodium azide caused the pharynx to be ejected from the body without affecting the rest of the worm, thus allowing them to monitor the process of pharynx regeneration. Blocking stem cell function by irradiation, or by RNA interference knockdown of stem cell-specific genes, prevented regeneration, which suggests that neoblasts have a crucial role in the regeneration of the pharynx.

**Figure 1.**
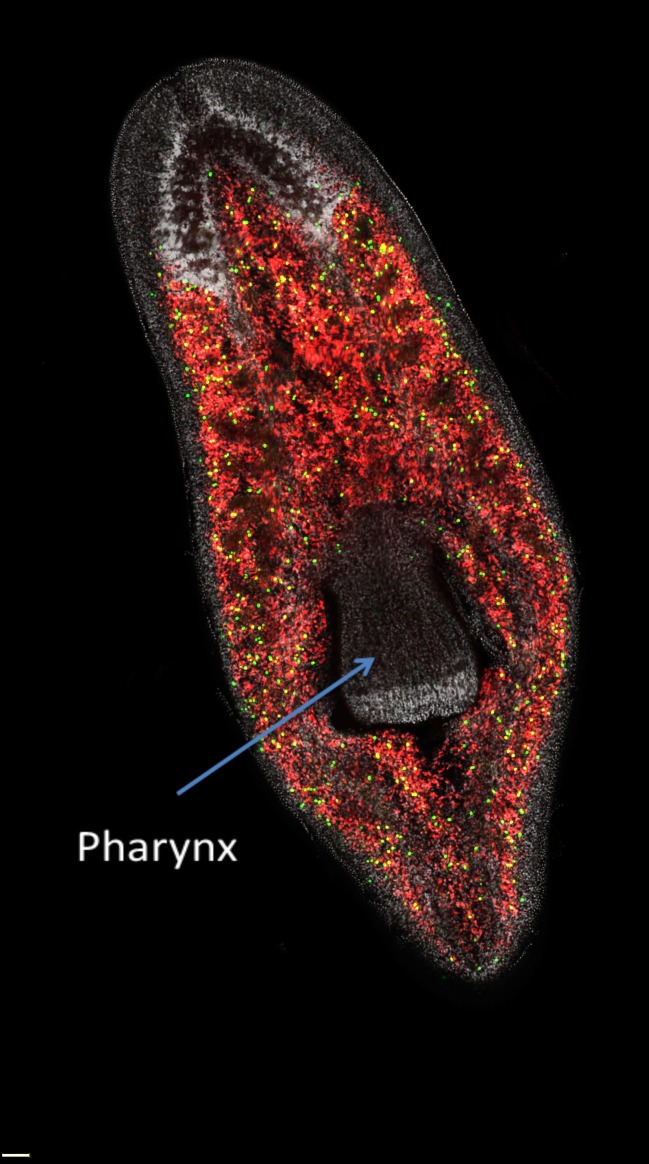
The distribution of neoblasts in a planarian. Neoblasts are found throughout the body but are excluded from the pharynx. The image distinguishes between neoblasts that are in the process of dividing to form new cells (green dots) and those that are not dividing (red dots) in a freshwater planarian called *Schmidtea mediterranea*. If the pharynx is removed, the actively dividing neoblasts and their progeny will migrate to the damaged area and begin organ regeneration.

Image courtesy of Alex Lin and Bret Pearson.

Local changes in gene expression at the wound site during the early phases of regeneration revealed that 356 genes were consistently expressed at higher levels than they were under normal conditions. The importance of each of these genes for regeneration was then tested by employing RNA interference knockdown to inhibit their expression, and using the ability of the planarian to feed as a simple way of measuring how well its pharynx had regenerated. Twenty genes affected feeding behaviour significantly; some affected general stem cell function; other genes affected feeding behaviour but did not affect tissue regeneration; and a small subset of genes specifically affected how the pharynx formed and functioned. The most severe but specific defects in pharynx formation were seen with the knockdown of the gene that encodes a Forkhead transcription factor called FoxA.

Forkhead proteins encoded by the *FoxA* gene family have important roles in specifying the tissues of the digestive system of invertebrates and vertebrates (Kaestner, 2010). In the simple sea anemone, FoxA marks the cells that will go on to form the pharynx (Fritzenwanker et al., 2004), while in the roundworm *C. elegans,* the equivalent of FoxA is key to specifying the different cell types found in the pharynx during development (Horner et al., 1998). In vertebrates, *FoxA* genes have switched from regulating the pharynx, the entry point to the intestine, to regulating the cells of the intestine itself (Ang et al., 1993).

In mammals, the FoxA family of transcription factors has also picked up other functions. These include specifying cell fate in the notochord and floorplate, structures that are crucial for axis formation in the developing embryo (Ang and Rossant, 1994; Weinstein et al., 1994), and controlling the function of adult neurons that produce dopamine (Stott et al., 2013). These divergent functions presumably relate to the fact that FoxA proteins are so-called pioneer factors. Pioneer factors bind to DNA at specific sites, opening up the chromatin structure in many different contexts (Zaret and Carroll, 2011). This means that they can function in combination with different transcription factors in different tissues. As a pioneer factor, FoxA may have played a fundamental evolutionary role in coordinating the formation of the structures needed to ingest and process food in multicellular organisms, and has then retained and expanded that role throughout evolution.

Planarian FoxA is expressed in the developing and the mature pharynx, and also in scattered neoblast cells around the pharynx that cluster at the site of amputation. In the absence of FoxA, neoblasts are still present, but they fail to migrate to the amputation site and initiate regeneration, and instead appear to be misdirected to other sites. So is FoxA primarily required for correct cell migration or for correct organ specification? In *C. elegans*, the equivalent of FoxA (which is called Pha-4) has been shown to bind directly to a wide range of genes that are required for pharynx structure and function (Gaudet and Mango, 2002), suggesting that it has a fundamental role in specifying pharynx cell fate. It will be very interesting to explore the direct targets of FoxA in planarians to see if it has a similar master regulatory function. Alternatively, it has been proposed that the evolutionarily conserved function of FoxA lies in regulating particular types of cell migration and rearrangement during development, rather than primarily in specifying cell fate (de-Leon, 2011). Since cell behaviour and cell fate are closely intertwined, it is hard to separate these functions. However, a more detailed comparison of the targets of FoxA at different stages of planarian regeneration with the corresponding targets in other species could reveal the fundamental roles of this important transcription factor.

## References

[bib1] Adler CE Seidel CW McKinney SA Sánchez Alvarado A 2014 Selective amputation of the pharynx identifies a FoxA-dependent regeneration program in planaria. eLife 3:e02238 doi: 10.7554/eLife.02238 PMC3985184 24737865

[bib2] Ang S-L Rossant J 1994 HNF-3beta is essential for node and notochord formation in mouse development. Cell 78:561–574 doi: 10.1016/0092-8674(94)90522-3 8069909

[bib3] Ang SL Wierda A Wong D Stevens KA Cascio S Rossant J Zaret KS 1993 The formation and maintenance of the definitive endoderm lineage in the mouse: involvement of HNF3/forkhead proteins. Development 119:1301–1315 8306889 10.1242/dev.119.4.1301

[bib4] de-Leon SB 2011 The conserved role and divergent regulation of foxa, a pan-eumetazoan developmental regulatory gene. Developmental Biology 357:21–26 doi: 10.1016/j.ydbio.2010.11.027 21130759 PMC3074024

[bib5] Fritzenwanker JH Saina M Technau U 2004 Analysis of forkhead and snail expression reveals epithelial-mesenchymal transitions during embryonic and larval development of *Nematostella vectensis*. Developmental Biology 275:389–402 doi: 10.1016/j.ydbio.2004.08.014 15501226

[bib6] Gaudet J Mango SE 2002 Regulation of organogenesis by the *Caenorhabditis elegans* FoxA protein PHA-4. Science 295:821–825 doi: 10.1126/science.1065175 11823633

[bib7] Horner MA Quintin S Domeier ME Kimble J Labouesse M Mango SE 1998 pha-4, an HNF-3 homolog, specifies pharyngeal organ identity in *Caenorhabditis elegans*. Genes & Development 12:1947–1952 doi: 10.1101/gad.12.13.1947 9649499 PMC316969

[bib8] Kaestner KH 2010 The FoxA factors in organogenesis and differentiation. Current Opinion in Genetics & Development 20:527–532 doi: 10.1016/j.gde.2010.06.005 20591647 PMC2943037

[bib9] Stott SR Metzakopian E Lin W Kaestner KH Hen R Ang SL 2013 Foxa1 and foxa2 are required for the maintenance of dopaminergic properties in ventral midbrain neurons at late embryonic stages. The Journal of Neuroscience 33:8022–8034 doi: 10.1523/JNEUROSCI.4774-12.2013 23637192 PMC6618950

[bib10] Wagner DE Wang IE Reddien PW 2011 Clonogenic neoblasts are pluripotent adult stem cells that underlie planarian regeneration. Science 332:811–816 doi: 10.1126/science.1203983 21566185 PMC3338249

[bib11] Weinstein DC Ruiz i Altaba A Chen WS Hoodless P Prezioso VR Jessell TM Darnell JE Jr 1994 The winged-helix transcription factor HNF-3 beta is required for notochord development in the mouse embryo. Cell 78:575–588 doi: 10.1016/0092-8674(94)90523-1 8069910

[bib12] Zaret KS Carroll JS 2011 Pioneer transcription factors: establishing competence for gene expression. Genes & Development 25:2227–2241 doi: 10.1101/gad.176826.111 22056668 PMC3219227

